# Sensitivity-enhanced three-dimensional and carbon-detected two-dimensional NMR of proteins using hyperpolarized water

**DOI:** 10.1007/s10858-020-00301-5

**Published:** 2020-02-10

**Authors:** Gregory L. Olsen, Or Szekely, Borja Mateos, Pavel Kadeřávek, Fabien Ferrage, Robert Konrat, Roberta Pierattelli, Isabella C. Felli, Geoffrey Bodenhausen, Dennis Kurzbach, Lucio Frydman

**Affiliations:** 1 Faculty of Chemistry, Institute for Biological Chemistry, University of Vienna, Währinger Straße 38, 1090 Vienna, Austria; 2 Department of Chemical and Biological Physics, Weizmann Institute of Science, Rehovot, Israel; 3 Department of Structural and Computational Biology, University of Vienna, Vienna BioCenter 5, 1030 Vienna, Austria; 4 CEITEC–Central European Institute of Technology, Masaryk University, Kamenice 5, 625 00 Brno, Czech Republic; 5 Laboratoire des biomolécules, LBM, Département de chimie, École normale supérieure, PSL University, Sorbonne Université, CNRS, 75005 Paris, France; 6 Magnetic Resonance Center and Department of Chemistry Ugo Schiff, University of Florence, Via L. Sacconi 6, 50019 Sesto Fiorentino, FI Italy

**Keywords:** Hyperpolarization, Dissolution-dynamic nuclear polarization (D-DNP), Direct ^13^C detection, 3D NMR, Non-uniform sampling, BEST-HNCO

## Abstract

Signal enhancements of up to two orders of magnitude in protein NMR can be achieved by employing HDO as a vector to introduce hyperpolarization into folded or intrinsically disordered proteins. In this approach, hyperpolarized HDO produced by dissolution-dynamic nuclear polarization (D-DNP) is mixed with a protein solution waiting in a high-field NMR spectrometer, whereupon amide proton exchange and nuclear Overhauser effects (NOE) transfer hyperpolarization to the protein and enable acquisition of a signal-enhanced high-resolution spectrum. To date, the use of this strategy has been limited to 1D and ^1^H-^15^N 2D correlation experiments. Here we introduce 2D ^13^C-detected D-DNP, to reduce exchange-induced broadening and other relaxation penalties that can adversely affect proton-detected D-DNP experiments. We also introduce hyperpolarized 3D spectroscopy, opening the possibility of D-DNP studies of larger proteins and IDPs, where assignment and residue-specific investigation may be impeded by spectral crowding. The signal enhancements obtained depend in particular on the rates of chemical and magnetic exchange of the observed residues, thus resulting in non-uniform ‘hyperpolarization-selective’ signal enhancements. The resulting spectral sparsity, however, makes it possible to resolve and monitor individual amino acids in IDPs of over 200 residues at acquisition times of just over a minute. We apply the proposed experiments to two model systems: the compactly folded protein ubiquitin, and the intrinsically disordered protein (IDP) osteopontin (OPN).

**Electronic supplementary material:**

The online version of this article (10.1007/s10858-020-00301-5) contains supplementary material, which is available to authorized users.

## Introduction

Nuclear magnetic resonance (NMR) spectroscopy is one of the principal tools for the investigation of the structural dynamics and structure–function relationships of proteins in solution (Theillet et al. 2016; Reichheld et al. 2017; Gil et al.^2013^; Yuwen and Skrynnikov 2014). A significant limitation of the method, however, is its low sensitivity, which can necessitate extensive signal-averaging. The acquisition of protein spectra thus often extends to hours or days, which can be prohibitive for the study of systems which are evolving rapidly or have limited stability in solution. We have recently explored the use of hyperpolarized water (Lipso et al. 2017) to boost signals in protein spectra. Hyperpolarized HDO produced using dissolution-dynamic nuclear polarization (D-DNP) can serve as a polarization reservoir, enhancing the ^1^H signals of sites undergoing chemical- or polarization exchange with the hyperpolarized solvent. This approach, which has been referred to as HYPEX (Kadeřávek et al. 2018) or HyperW (Szekely et al. 2018), is applicable to a broad range of aqueous systems without requiring any special properties or chemical modification of the molecules under study, and makes it possible to achieve over 100-fold signal enhancements in spectra of folded or intrinsically disordered proteins (Kadeřávek et al. 2018; Szekely et al. 2018; Kurzbach et. al. 2017; Olsen et al. 2016; Kim et al. 2017; Ragavan et al. 2017; Wang and Hilty 2019; Doll et al. 2012; Viennet et al. 2016).

In the present study, we expand this approach to include three-dimensional (3D) and ^13^C-detected 2D experiments (Gil et al. 2013), which we demonstrate using two representative protein systems: the compactly folded model protein ubiquitin (Vijay-Kumar and Bugg 1987; Hamilton et al. 2000), and the intrinsically disordered protein (IDP) osteopontin (OPN), and its complex with its ligand heparin (Kurzbach et al. 2013; Rodrigues et al. 2007). Using the proposed experiments, we obtain hyperpolarized ‘fingerprints’ of the two proteins, and are also able to detect a minor OPN population that would likely have remained undetected by conventional thermal equilibrium NMR spectroscopy.

## Results and discussion

The hyperpolarized HDO-based buffers that are at the heart of this experimental strategy were produced using D-DNP systems operating at ~ 1.2 K and magnetic fields of either 3.35 T (Oxford HyperSense™) or 6.7 T (Bruker BioSpin). The general procedure is as follows (for details see the Experimental section): (i) Hyperpolarization of a frozen H_2_O solution containing a paramagnetic polarization agent and a cryo-protectant is achieved by means of off-resonance microwave irradiation. (ii) After building up the polarization for 2–3 h, the hyperpolarized H_2_O is dissolved with a burst of superheated neat or buffered D_2_O to produce a mixture containing 1–4% HDO (depending on the system and experimental parameters), which is then injected in 1–3 s into an NMR tube waiting in a high-field detection spectrometer, where it mixes in-situ with the target protein solution. (iii) Chemical and ‘magnetic’ exchange processes (transient Overhauser effects) between hyperpolarized HDO and the protein transfer ^1^H-hyperpolarization to the protein, thereby selectively enhancing NMR signals of residues with favorable solvent interactions (Kadeřávek et al. 2018; Szekely et al. 2018; Kurzbach et al. 2017; Olsen et al. 2016; Kim et al. 2017).

Signal intensities for all experiments are tabulated in the Supplementary Information. In most cases, even after extensive signal averaging, the signals in thermal equilibrium spectra for the D-DNP samples were too weak for a reliable determination of the enhancement factor ‘ε’ (often defined as the ratio between per-scan signal amplitudes in the hyperpolarized spectrum and the corresponding spectrum acquired after return of the system to thermal equilibrium). Therefore, to quantify the signal intensities obtained with hyperpolarized HDO, we instead report signal-to-noise ratios (SNR) for the hyperpolarized spectra. (As an approximate lower bound for ε, one can thus assume that ε ≥ SNR for instances where the corresponding reference signal remains below the limit of detection).

### Hyperpolarized high-resolution ^13^C-detected 2D NMR

In previous work, we demonstrated how solvent hyperpolarization can be combined with rapid 2D ^1^H-^15^N HMQC spectroscopy (Schanda et al. 2005) of folded proteins and small IDPs (Kadeřávek et al. 2018; Szekely et al. 2018; Kurzbach et al. 2017; Olsen et al. 2016; Kim et al. 2017). However, analysis of proton-detected hyperpolarized 2D spectra of larger proteins or IDPs is sometimes challenging, as signal overlap and broadening due to exchange, radiation damping, and paramagnetic relaxation may hinder spectral analysis. To address these limitations, we expand our approach here to include ^13^C-detected ^13^C-^15^N correlation experiments (H^N^-CON, see pulse sequence in Fig. S1) (Gil et al. 2013; Bertini et al. 2011). In these experiments, chemical and magnetic exchange transfer proton hyperpolarization from HDO to protein backbone amide (and side-chain) sites. The amide hyperpolarization is then transferred by selective INEPT to the neighboring ^15^N nuclei, and, after an ^15^N evolution period, transferred onward to the adjacent ^13^CO spins for detection (Gil et al. 2013). The amide proton polarization is thus continuously replenished in successive scans by the hyperpolarized HDO pool, while ^1^H-detection is avoided.

Direct ^13^C detection offers the added advantage that the chemical shift dispersion in the ^13^C dimension is much larger compared to ^1^H, while at the same time the penalties associated with ^1^H detection are reduced.

#### Application to a folded protein: Ubiquitin

**Fig. 1:**
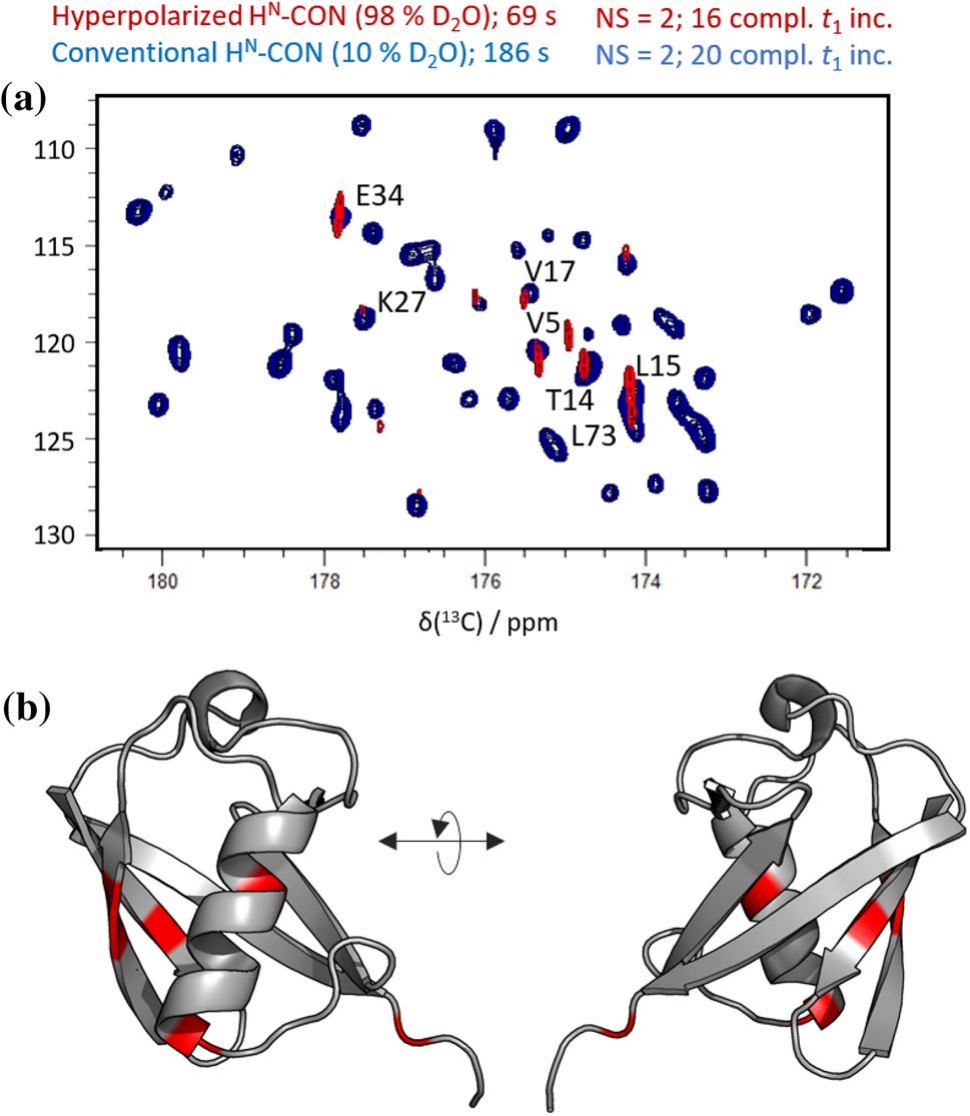
**a** Hyperpolarized H^N^-CON correlation spectrum of ubiquitin in 98% deuterated buffer (red signals), superimposed on the corresponding thermal equilibrium spectrum obtainedin 10% deuterated buffer (blue peaks). The assignments denote residue {*i*}, for each {*i, i* − 1} ^15^N-^13^C cross peak. **b** Ubiquitin crystal structure (PDB code 1ubq), with the subset of residues detected in experiments with hyperpolarized HDO highlighted in red. These consist primarily of residues undergoing rapid solvent exchange, but also include several cases where the enhanced polarization has migrated to adjacent core residues. (Signal intensities are tabulated in Supplementary Table S1.)

A hyperpolarized H^N^-CON spectrum obtained for ubiquitin via this strategy is shown in Fig. 1a (red), overlaid with a conventional H^N^-CON spectrum of ubiquitin obtained at thermal equilibrium in a 90:10% H_2_O:D_2_O phosphate buffered solution (blue). The hyperpolarized spectrum was acquired in 69 s at 37 °C. Both spectra were obtained in a magnetic field of 14.1 T (600 MHz).

Only if chemical and magnetic exchange from HDO is sufficiently fast will enhanced signals be observed (Kadeřávek et al. 2018; Szekely et al. 2018; Kurzbach et al. 2017; Olsen et al. 2016; Kim et al. 2017; Nucci et al. 2011; Otting 1997; Modig et al. 2004). In particular, the signal enhancementfor an amide site should be increased by faster exchange rates on the one hand, as replenishment of hyperpolarized amide protons between successive scans is improved, while on the other hand the improvement will be attenuated by losses due to proton exchange and relaxation during evolution delays and detection. Thus, as expected, the hyperpolarized spectrum contains a reduced subset of the signals seen in the corresponding conventional spectrum. Figure 1b maps the observed signals onto the crystal structure of ubiquitin, revealing that some residues in the hydrophobic core are also detected, indicating that hyperpolarization may in some cases be transferred from the solvent-exposed surface to the protein’s inner core.

The set of peaks observed depends on many variables, in particular on the overall SNR characteristic of the experiment in question, the delay between successive detections, and as noted above, the hyperpolarization transfer efficiency through proton exchange, nuclear Overhauser effects (NOE), or exchange-relayed NOE. While the latter three polarization transfer pathways do not critically depend on the pulse sequence or nucleus used for detection, the SNR of course does. However, if the underlying HYPEX/HyperW exchange and NOE polarization transfer mechanisms are operating as in our earlier proton-detected work (Kadeřávek et al. 2018), one should anticipate a qualitative agreement between the set of residues observed in the hyperpolarized proton-detected ^1^H/^15^N HMQC spectrum reported there and in the ^13^C-detected spectrum we obtain here. And indeed, the H^N^-CON results confirm these expectations, as all peaks observed with H^N^-CON detection were also observed in the hyperpolarized HMQC. Note that in the H^N^-CON, we do not see all of the peaks seen in the HMQC spectrum; we see instead only the set of peaks which showed the highest SNR in the proton-detected experiment. The reduction in the number of detected residues is due to several factors, such as the additional coherence transfer steps in the H^N^-CON experiments and the intrinsically lower sensitivity of ^13^C detection.

Moreover, as seen in Fig. 1a, despite the rapid injections and brief experimental durations used, the linewidths in the directly-detected dimension of the hyperpolarized H^N^-CON (10–15 Hz) are comparable to or narrower than those in its conventional counterpart, and approximately half of those obtained previously using ^1^H-detection (25–30 Hz in hyperpolarized ^1^H/^15^N HMQC) (Kadeřávek et al. 2018), indicating that ^13^C-detection reduces radiation damping and/or decoherence due to rapid proton exchange and paramagnetic relaxation – factors that limited resolution in earlier proton-detected hyperpolarized studies of protein systems (Kurzbach et al. 2017).

#### Application to an IDP: Osteopontin and its complex with heparin

**Fig. 2:**
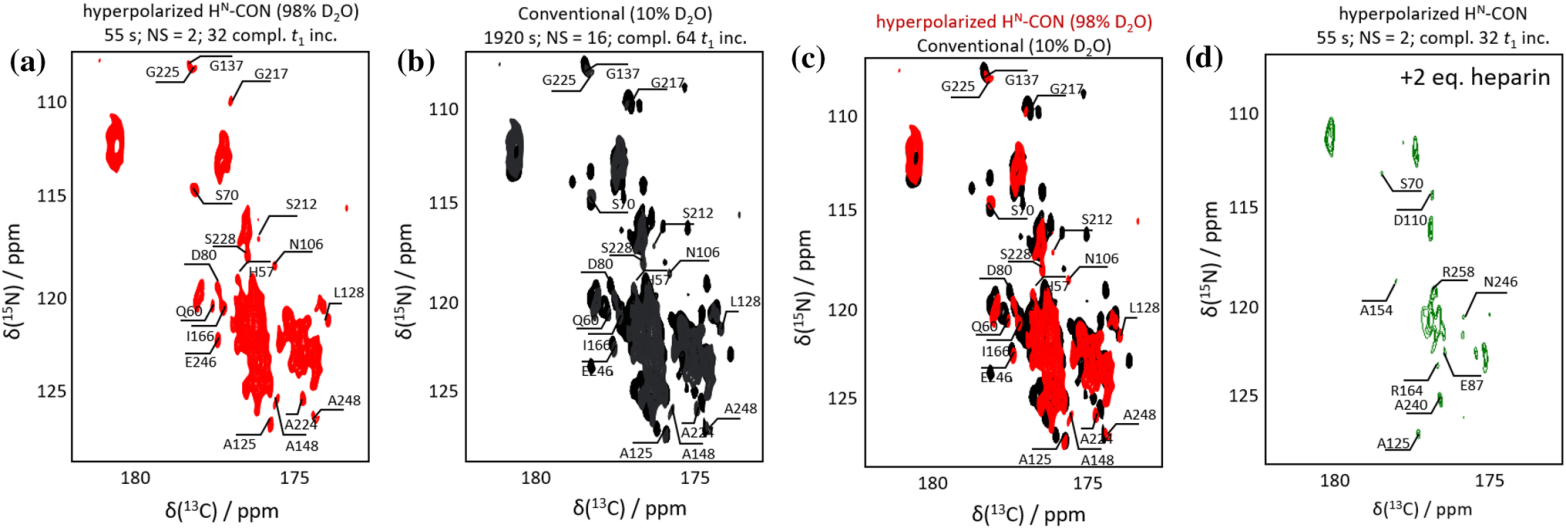
**a** Hyperpolarized H^N^-CON correlation spectrum of osteopontin detected in 55 s using hyperpolarized HDO, in ca. 98% deuterated buffer, with assignments of a few well-resolved residues. **b** conventional reference spectrum acquired in 10% deuterated buffer. **c** Overlay of the hyperpolarized spectrum (red) and the reference spectrum (black). **d** Hyperpolarized H^N^-CON correlation spectrum of OPN in the presence of 2 equivalents of heparin, with assignments of some well-resolved residues. (Signal intensities are tabulated in Supplementary Tables S2 and S3.)

Figure 2a displays a hyperpolarized ^13^C-^15^N spectrum of a 220-residue truncation mutant of OPN. Figure 2b shows a thermal equilibrium reference spectrum collected in a 10% deuterated buffer, and Fig. 2c shows a comparison of the hyperpolarized and reference spectra. The hyperpolarized spectrum was obtained in 55 s at 37 °C and pH 7.4 at 14.1 T (600 MHz for ^1^H). As for ubiquitin, the hyperpolarized and conventional H^N^-CON spectra show nearly identical linewidths. In contrast to the folded ubiquitin, however, a markedly larger fraction of the peaks observed in the conventional spectrum of OPN are also present in the hyperpolarized spectrum. This is not surprising, given the greater solvent exposure expected for an IDP, which would be consistent with particularly efficient chemical exchange and polarization transfer.

A strikingly different spectral response is observed when OPN is monitored after mixing with its ligand heparin (Clemente 2016). The heparin interaction site spans OPN residues 100 to 180. Upon binding of the large 17 kDa ligand, much of the IDP is in direct contact with heparin, and thus likely becomes somewhat shielded from the solvent. Indeed, when probed by hyperpolarized H^N^-CON after addition of heparin, the number of detected OPN peaks drops significantly, and the subset of observed residues changes markedly as well (Fig. 2d).

Due to signal overlap, numerous peaks observed in both the conventional and hyperpolarized spectra of OPN cannot be assigned with 2D HMQC or H^N^-CON data alone, making it difficult to determine the true spectral ‘binding footprint’ for this system*.* However, the fact that the pronounced drop in observed signals is not restricted to the binding site suggests that screening by the ligand alone is not the only determinant of changes in polarization transfer efficiency in the complex. Complex formation would also be expected to affect solvent exposure and polarization transfer of newly-sequestered residues substantially, perhaps resulting in a sparse enhancement pattern not unlike that seen for ubiquitin.

### Hyperpolarized three-dimensional NMR

While the improvements in resolution provided by the hyperpolarized H^N^-CON relative to hyperpolarized ^1^H-^15^N HMQC experiments can be substantial, particularly for samples such as OPN (Mateos et al. 2019), in many instances 2D NMR cannot reduce signal overlap sufficiently to permit residue-specific analysis. To further reduce spectral crowding and to widen the scope of our approach, we therefore implemented hyperpolarized 3D ^1^H-^13^C-^15^N correlation experiments—in particular, the BEST-HNCO experiment shown in Fig. S2 (Lescop et al. 2007).

#### Methodological considerations

Given a final solvent deuteration level of 96–98% after dissolution and mixing, typical proton hyperpolarization lifetimes in these experiments are less than 2 min (*T*_1_ (^1^H) ≈ 20 s). To permit detection of two indirectly encoded dimensions under this time constraint, the use of non-uniform sampling (NUS) (Zawadzka-Kazimierczuk et al. 2012; Mayzel et al. 2014) is mandatory. In the present case, after empirical optimization, 9% NUS was chosen to provide a compromise between spectral resolution along ω_1_ (^13^C) and ω_2_ (^15^N), while allowing us to complete the 3D acquisitions within the time restriction of the HDO polarization lifetime. Poisson gap sampling (Hyberts et al. 2012) was used to generate NUS schedules, and a total of 96 FIDs were recorded in 65–75 s for a nominal digital resolution of 44 × 89 × 352 Hz in ω_3_, ω_2_ and ω_1_ respectively. The hyperpolarized HNCO spectra presented here were acquired in 65 s (OPN + Heparin) or 75 s (ubiquitin, OPN) at 18.8 T, in 96% deuterated buffer, at pH 7.4 and 37 °C.

The full 3D spectra were reconstructed using hmsIST (Hyberts et al. 2012) in NMRPipe (Delaglio et al. 1995), and all spectra were cross-checked against conventional thermal equilibrium spectra to identify potential spectral reconstruction artifacts. While many alternative NUS strategies are available (Kazimierczuk et al. 2009, 2010), the HMS implementations of sampling and reconstruction were chosen for the proof-of-concept experiments described here, as they are readily available to the NMR community and robust in our hands (https://gwagner.med.harvard.edu/intranet/hmsIST/).

#### Ubiquitin

**Fig. 3:**
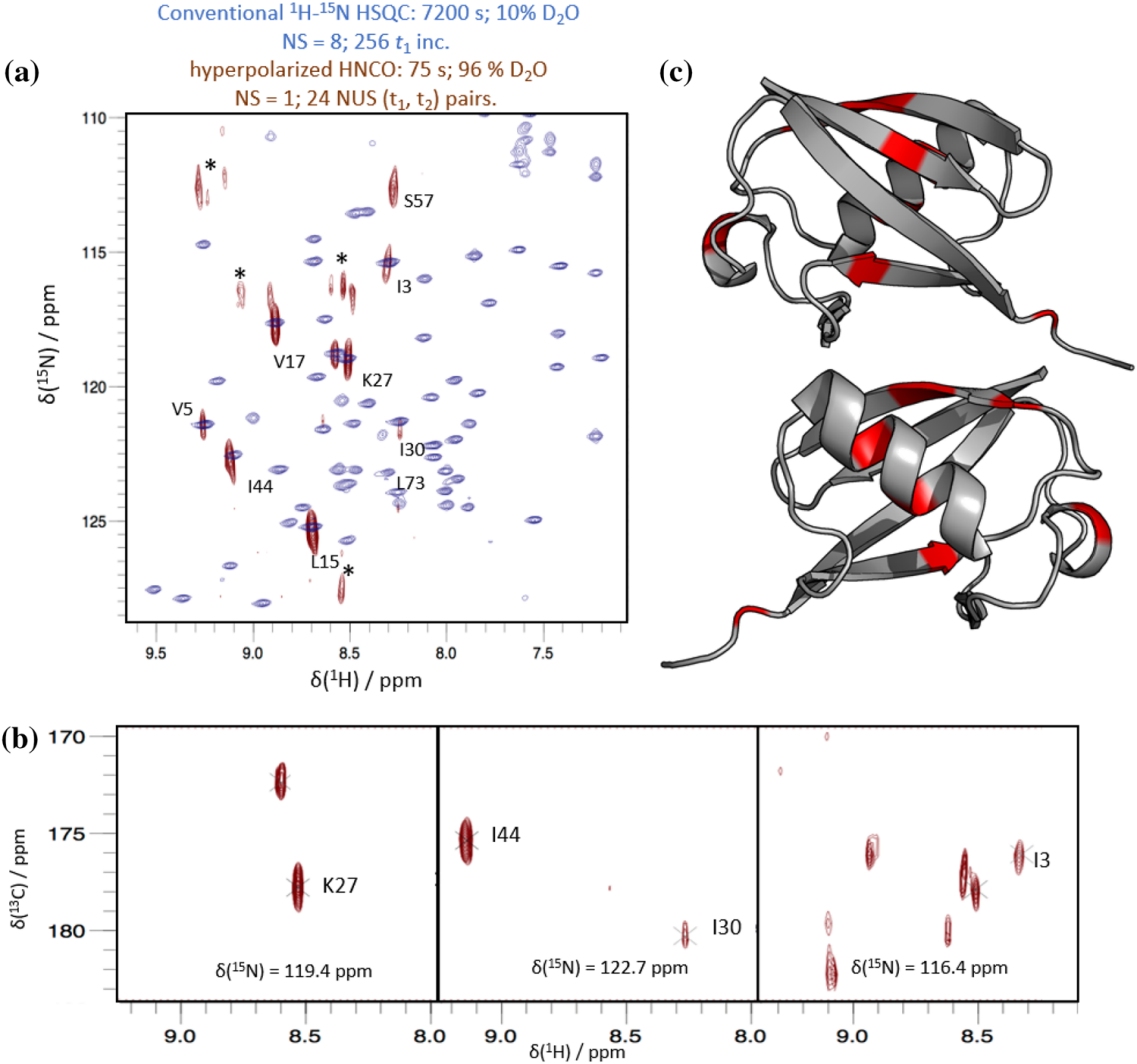
**a** Overlay of a conventional ^1^H-^15^N HSQC spectrum of ubiquitin obtained in 2 h (blue) and the projection onto the ^1^H-^15^N plane of a selectively hyperpolarized 3D HNCO spectrum of ubiquitin obtained in 75 s using NUS (red). Assignments for ubiquitin at pH 7.4 were based upon pH titration experiments (see Fig. S10). Asterisks denote possible reconstruction artifacts. **b** Planes representative of the selectively hyperpolarized HNCO. **c** Selectively hyperpolarized residues (red) mapped onto the crystal structure (PDB code 1ubq) of ubiquitin. (Signal intensities are tabulated in Supplementary Table S4.)

As for the selectively hyperpolarized H^N^-CON experiments described above, we first tested the hyperpolarized HNCO using ubiquitin as a representative folded protein. Figure 3a displays the projection of the ^1^H-^15^N plane of a hyperpolarized 3D HNCO spectrum collected in just over one minute overlaid onto a conventional ^1^H-^15^N 2D HSQC of ubiquitin detected in 2 h (in 90:10 H_2_O:D_2_O, at pH 7.4 and 37 °C). Representative planes of the hyperpolarized HNCO spectrum are shown in Fig. 3b. While the conventional 2D reference spectrum has better resolution than its hyperpolarized 3D counterpart, as expected, the subset of peaks obtained in the hyperpolarized HNCO spectrum are in good agreement with their counterparts in the conventional 2D spectrum, and the linewidths in the NUS 3D are near the nominal resolution expected for an equivalent ‘fully-sampled’ 75 s acquisition (^15^N nominal resolution: 89 Hz (~ 1.1 ppm); ^15^N observed resolution: 1–2 ppm). Figure 3c maps the detected residues (in red) onto the ubiquitin crystal structure. This set of detected signals corresponds to the one obtained by the hyperpolarized H^N^-CON (Fig. 1b), as all peaks observed in the H^N^-CON are also observed in the HNCO. Notably, as was seen previously in the hyperpolarized HMQC of ubiquitin (Kadeřávek et al. 2018), significant enhancements are observed not only for solvent-exposed surface residues, but also for several residues in the protein core.

#### Osteopontin and its ligand heparin

**Fig. 4:**
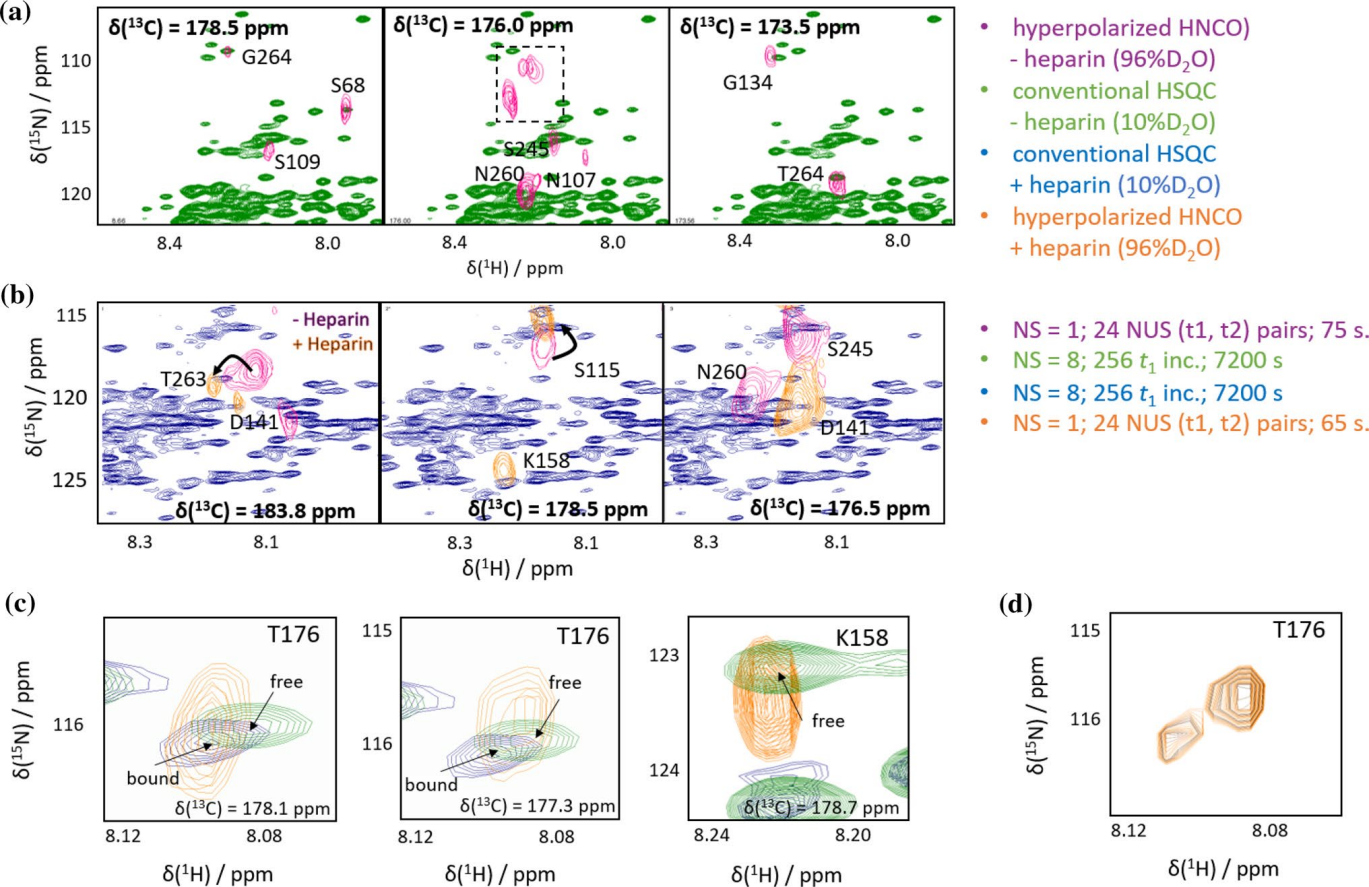
Detection of heparin binding to OPN: **a** Overlay of a conventional thermal equilibrium 2D ^1^H-^15^N HSQC spectrum of free OPN in 10% deuterated buffer (green) with three planes from a hyperpolarized 3D NUS HNCO in 96% deuterated buffer (magenta). The dashed box indicates signals of arginine side chains that are folded in both the ^13^C- and the ^15^N dimension. **b** Overlay of the conventional 2D ^1^H-^15^N HSQC spectrum of OPN in the presence of 2 equivalents of heparin (blue) and three hyperpolarized 3D NUS HNCO planes (orange: with heparin; magenta: without heparin). **c** Superpositions of three zoomed planes from the hyperpolarized HNCO spectrum in the presence of heparin (orange), and conventional ^1^H-^15^N HSQCs of OPN in the presence (blue) and absence (green) of heparin. Both ligand-free and ligand-bound states are detectable in the HNCO spectrum for residue T176, which is located in the binding site and experiences a chemical shift upon heparin binding. The signal of the heparin-bound form of OPN can be seen in the δ(^13^C) = 178.1 ppm HNCO plane (left), where the bound-form peak (in yellow) coincides with that of the heparin-bound reference (blue), while the heparin-free form is observed in the δ(^13^C) = 177.3 ppm HNCO plane (center), where the signal now coincides with the heparin-free reference (green). For residue K158 (also located in the binding site; right), only the signal of the free form is observed in the presence of heparin, whereas in the bound form this residue is broadened beyond detection in both conventional and hyperpolarized spectra. (Signal intensities are tabulated in Supplementary Tables S5 and S6.) **d** Superposition of the zoomed ^1^H-^15^N planes of the HNCO shown at left and center in (**c**) (at δ(^13^C) = 177.3 ppm and δ(^13^C) = 178.1 ppm)), at different cut-off levels showing the two distinct peaks which can be discerned for residue T176 in the 3D experiment

Figure 4a, b shows representative planes of the hyperpolarized HNCO spectra of the heparin-free and -bound states of OPN (magenta and orange peaks, respectively), overlaid on the corresponding conventional thermal equilibrium HSQC spectra (green and blue, respectively). Figure 4c illustrates the residue-selectivity of the hyperpolarization in the presence and absence of heparin. The signal overlap in conventional 2D spectra of this system would make a transfer of assignments and spectral analysis particularly challenging (see also the Supporting Information). Here, extension of hyperpolarized experiments to encompass a third spectral dimension provides a significant improvement in resolution, and is also able to highlight several features of the OPN-heparin interaction.

Notably, in the presence of 2 equivalents of the ligand, traces of a second small population were seen, which we attribute to the unbound IDP. This could not be observed in conventionally detected spectra of the complex. This is illustrated in Fig. 4c, d, where spectral signatures of both the free and bound forms of OPN are detected for residue T176, while in the corresponding conventional spectrum, only the bound form is seen. If we assume that free OPN is not screened by the ligand, and thus more readily enhanced, solvent-based hyperpolarization would be expected to be particularly effective in highlighting the small population of free OPN even in the presence of a much larger population of the bound state. For residue K158 this effect is indeed observed, as only the free form is seen in the presence of heparin. While the pH and temperature used here (7.4, 37 °C) differ from those used in previous studies of this system, which may affect the visibility of certain residues, in any case the ability to detect such small populations should prove valuable for the characterization of sparsely-populated states.

Due to substantial overlap, the resolution in hyperpolarized 1D or 2D spectroscopy would not be sufficient to distinguish these two signals (see Fig. S9 in the Supporting Information for representative 1D projections). In the 3D experiments, however, these peaks are well isolated from other resonances and thus could be unambiguously assigned. As can be seen in Fig. 4c, the ^15^N chemical shifts of the two T176 signals are in good but not perfect agreement with those observed for the two peaks in this region of the static reference spectra. While we cannot exclude the possibility that alternative OPN conformations giving a similar but slightly shifted spectral signature could be present in the sample, the presence of distinct T176 signals in the 3D spectrum is consistent with the presence of both a bound and an unbound OPN species following heparin addition. Thus, by combining the improved sensitivity provided by hyperpolarized water with the improved resolution afforded by 3D detection, signatures of even small populations of minor states can be discerned.

## Conclusions

The present study demonstrates how, by combining the use of hyperpolarized water with rapid 3D- and ^13^C-detected 2D experiments, we are able to take advantage of the enhanced sensitivity available in earlier hyperpolarized 1D and 2D ^1^H-^15^N HMQC experiments while providing the improved resolution needed to extend the method to larger proteins and IDPs.

Hyperpolarized HDO-based experiments such as those introduced here typically produce spectra showing non-uniform ‘hyperpolarization-selective’ signal enhancements. Many factors need to be considered when trying to predict the likelihood that the intensity of a given residue will be boosted by means of hyperpolarized solvents, most importantly the proton exchange rate, but also the contributions of direct- or exchange-relayed NOE (Kadeřávek et al. 2018). As noted above, the improvement in signal intensity for an amide site is increased by faster exchange rates on the one hand, as replenishment of hyperpolarized amide protons between successive scans is improved, while on the other hand the improvement is attenuated by losses due to proton exchange and relaxation during transfer, evolution, and detection. Moreover, the efficiency of the hyperpolarization also depends on the details of local solvation, on the ionic strength, and on the polarization and relaxation rate of the HDO, sothat reliable predictions are challenging.

However, it is already clear that the use of hyperpolarized water to boost protein signals not only provides a more sensitive detection, but also achieves a ‘hyperpolarization-selective labeling’, carrying valuable insight about the rates of chemical and magnetic (NOE) polarization exchange between solvent and protein.

It should also be noted that the experiments presented here suffer a substantial SNR penalty as a result of the dilution of the hyperpolarized water during the dissolution process: in contrast to the 90% H_2_O buffers commonly used in standard NMR measurements, the final H_2_O fractions available to populate the protein sites are typically reduced to 2–4% after mixing of the hyperpolarized HDO with the waiting sample. Most of the protein is thus not visible due to deuteration of the amide protons (and consequently, in most cases we could not detect any protein signals for the samples measured here after their return to thermal equilibrium.) The required initial protein concentrations are thus comparable to those used in standard experiments. Until dilution during dissolution can be reduced, this will remain a primary limitation of the method.

For exchanging sites however, the loss in signal intensity due to limited proton availability in the solvent fraction is compensated due to the large enhancements (often greater than 500-fold) carried by the incoming hyperpolarized HDO, making them selectively visible, and doing so very rapidly when exchange is favorable. A key benefit of the reduced acquisition times demonstrated here should thus be significantly improved time-resolution for monitoring of rapid processes involving growth or evolution of small populations of transient species which can be made visible and resolvable using dissolution DNP. While it has been shown here that both folded and intrinsically disordered proteins are amenable to the method, IDPs in particular suffer from well-known resolution limitations when studied by ^1^H-^15^N 2D correlation methods. Hyperpolarized ^13^C-detected experiments can reduce these penalties, by providing improved spectral dispersion and reducing the broadening effects of rapid solvent exchange. Thus, while the H^N^-CON may not be particularly informative for samples like ubiquitin, which was used here to provide a proof of concept, for systems like OPN where extensive broadening renders attempts to resolve hyperpolarized ^1^H/^15^N correlations difficult or even impossible, the experiment should prove valuable, as it can provide useful site-specific information when alternative D-DNP-enhanced experiments would otherwise be unavailable. Furthermore, the extensive solvent exposure of IDPs facilitates good water-based enhancements, while preserving access to site-specific information about exchange. These combined resolution and sensitivity benefits are made available in hyperpolarized 2D H^N^-CON NMR experiments.

Hyperpolarized 3D experiments were also shown here to be feasible via the use of NUS, which made 3D acquisitions compatible with the limited lifetime of hyperpolarized HDO. The power of the resulting method was demonstrated by its ability to discern residues involved in the binding undergone by a relatively large IDP like osteopontin. It also permitted the detection of small protein populations whose characterization would have been challenging or impossible by other NMR methods.

We expect the proposed experiments to be particularly useful for the study of phenomena such as post-translational modifications (Bah and Forman-Kay 2016), as such protein modification sites are frequently solvent-exposed and hence particularly well suited for investigation by water-derived hyperpolarized experiments.

## Material and methods

### Dissolution and polarization conditions

For the hyperpolarized H^N^-CON experiments, 150 μL of a 10 mM solution of 4-amino-TEMPO in an 85:15 v/v mixture of water and glycerol was vitrified in liquid helium. The glassy sample was positively hyperpolarized for ~ 3 h at ~ 1.16 K in a magnetic field of 3.35 T by partial saturation of the EPR spectrum at 94.195 GHz (Oxford HyperSense™, Weizmann Institute). Dissolution was achieved with a burst of 2.8 mL deuterated 20 mM phosphate buffered saline (PBS) at 180 °C under 0.9 MPa. The sample was subsequently propelled to the nearby detection NMR spectrometer operating at 14.1 T (600 MHz ^1^H NMR) and 37 °C within 1.5 s with He gas (Olsen et al. 2016), where it was mixed with 150 μL of target protein solution. Transfer and injection of hyperpolarized HDO was performed using an automated high-pressure injection system optimized to deliver reproducible volumes of solvent to the waiting protein solution while suppressing bubble and foam formation during mixing (Szekely et al. 2018; Olsen et al. 2016; Katsikis et al. 2015). NMR experiments were then triggered after a 3 s delay for mixing and settling.

For the hyperpolarized HNCO experiments, 180 μL of a 15 mM TEMPOL solution in an 85:15 v/v mixture of water and glycerol was vitrified in liquid helium. The glassy sample was positively hyperpolarized at 1.2 K in a magnetic field of 6.7 T by partial saturation of the EPR spectrum at 187.7 GHz (Bruker Biospin, ENS Paris). Under these conditions, the hyperpolarization build-up rates were found to be *R*_*build-up*_ = (4.8 ± 0.1) x 10^−5^ s^−1^, hence hyperpolarization buildup times of 3 h were used. Dissolution was achieved with a burst of 5 mL D_2_O at 180 °C under 1.05 MPa. The sample was subsequently propelled to a detection NMR spectrometer operating at 18.8 T (800 MHz for ^1^H) and 37 °C within 1.3 s with He gas under 0.7 MPa through a “magnetic tunnel” (Milani et al. 2015) maintaining a constant magnetic field of 0.9 T over a distance of approximately 4 m. The delay for mixing with the target protein solution (150 μL) and settling of turbulence prior to initiation of the NMR experiments was again 3 s.

### NMR spectroscopy

^1^H and ^13^C chemical shifts were referenced to DSS, ^15^N chemical shifts were referenced according to Markley et al. (1998).

### H^N^-CON experiments

The H^N^-CON experiments were implemented as described by Felli and co-workers (Gil et al. 2013). All H^N^-CON spectra were recorded on a 14.1 T (600 MHz) Bruker Avance III^®^ NMR spectrometer equipped with a 5 mm Prodigy^®^ cryoprobe. Bandwidths and offsets of shaped pulses were optimized empirically to maximize excitation of amide protons while minimizing unwanted depletion of the hyperpolarized HDO pool, as well as radiation damping effects that would result if the hyperpolarized water resonance were excited. As the HDO resonance in hyperpolarized water experiments is typically quite broad, the bandwidths of the proton excitation and refocusing pulses are more tightly constrained than in experiments at thermal equilibrium. In our hands, a good compromise for avoiding excitation of the water resonance while still exciting the target H^N^ amide protons of interest was obtained by applying the proton excitation and refocusing pulses at 9.5–10.5 ppm while increasing their bandwidths to ~ 6 ppm. Selective ^1^H excitation and refocusing were achieved using PC9 (Kupce and Freeman 1994) (bandwidth 6.25 ppm, duration 2001 µs), and REBURP (Geen and Freeman 1991) (bandwidths 6.0–6.05 ppm, durations 1600–1614 µs) pulses, respectively. Selective proton pulses were centered at 9.75 ppm (OPN) or 10.5 ppm (ubiquitin).

Selective ^13^C excitation and refocusing were achieved using Q5 (bandwidth 136 ppm, duration 300 µs), and Q3 (bandwidth 115 ppm, duration 199 µs) pulses (Emsley et al. 1990). Selective ^13^C pulses were centered at 173 and 54 ppm for CO and Cα, respectively. IPAP acquisition was used to suppress CO-Cα couplings during acquisition. Non-selective ^15^N 90° pulse durations were 31.5 µs (ubiquitin) and 32.9 µs (OPN).

The spectral widths used were 19.9 and 26.3 ppm, respectively, for ^13^C and ^15^N for ubiquitin, and 30.0 and 26.3 ppm for OPN.

For ubiquitin, the acquisition time in the direct dimension was 85 ms, the recycle delay was 350 ms, and the total time per transient was 537 ms. A total of 64 FIDs (in-phase plus anti-phase) were collected, which were combined to give 16 complex increments. Two scans were collected per increment, to give a total experimental duration of 69 s.

For OPN, the acquisition time in the direct dimension was 56 ms, the recycle delay was 50 ms, and the total time per transient 210 ms. A total of 128 FIDs (in-phase plus anti-phase) were collected, which were combined to give 32 complex increments. Two scans were again collected per increment, to give a total experimental duration of 55 s.

Reference H^N^-CON spectra for ubiquitin and unbound OPN were collected at thermal equilibrium using experimental parameters matched to the corresponding hyperpolarized experiments, but with longer experimental durations, and using 2 mM samples in 90% protonated PBS buffer. For the ubiquitin reference, the number of scans was again 2, the recycle delay was increased to 1 s, and the number of t_1_ increments collected was increased to 80 (20 complex t_1_ increments), to give a total experimental duration of 3 min 6 s. For OPN, the number of scans was increased to 16, the recycle delay was increased to 300 ms, and the number of t_1_ increments collected was doubled to 256 (64 complex t_1_ increments), to give a total experimental duration of 32 min.

### HNCO experiments

^1^H, ^13^C, ^15^N BEST HNCO spectra were recorded on a wide-bore 18.8 T Bruker Avance III HD NMR spectrometer equipped with a TXI probe, using the Bruker library pulse sequence ‘b_hncogp3d’ (modified to generate shaped ^1^H pulses directly from input values of offsets, flip-angles, and bandwidths) (Lescop et al. 2007). As for the H^N^-CON experiments, shaped ^1^H pulse bandwidth/offset combinations were again optimized empirically.

Selective ^1^H pulses were PC9 (bandwidth 4.17 ppm, duration 2251 µs), REBURP (bandwidth 4.85 ppm, duration 1498 µs), EBURP2 and EBURP2tr (bandwidths 4.3 ppm, durations 1439 µs), and BIP720, 50, 20.1 (150 µs) (Smith et al. 2001). Except for the BIP pulse (set on resonance at 4.7 ppm), all ^1^H selective pulses were centered at 10.5 ppm (ubiquitin and OPN) or 9.9 ppm (OPN-heparin).

Selective ^13^C pulses were G4 and G4tr (bandwidth 125 ppm, duration 308 µs), and Q3 (bandwidth 82 ppm, duration 210 µs) (Emsley et al. 1990). Selective ^13^C pulses were centered at 173 ppm for CO, or 53.2 ppm for Cα. Non-selective ^15^N 90° pulse durations were 39.75 µs.

Spectral widths acquired were 13.95 ppm for ^1^H, 35.0 ppm for ^15^N, and 14.0 ppm for ^13^C. Carrier frequencies were 4.7 ppm (^1^H), 173.5 ppm (^13^C) and 117 ppm (^15^N). Poisson-gap NUS at 9.4% sampling was used, with sampling schedules generated using the HMSist ‘nusPGSv3′ AU macro (Hyberts et al. 2012). Twenty-four {Δ*t*_1_, Δ*t*_2_} increment pairs were collected, to represent the 32 × 8 complex pairs (TD = 512 × 64 × 16) of the corresponding fully-sampled HNCO experiment (or 512 × 32 × 32, and 24 increment pairs, for the OPN + heparin sample). The acquisition time in the direct dimension was 23 ms, the recycle delay 600 ms (500 ms for OPN + heparin), and the total time per transient 664 ms, to give a total experimental time of 75 s (65 s for OPN + heparin). The full 3D spectra were reconstructed using the hmsIST (Hyberts et al. 2012) add-on in NMRPipe (Delaglio 1995) and analyzed with the Sparky (Goddard and Kneller 2008) program package.

Reference HSQC spectra were collected at thermal equilibrium using the standard Bruker sequence ‘fhsqcf3gpph’ (Mori et al. 1995). Signals were averaged over 16 scans, and 256 real and 256 imaginary *t*_1_ points (Δ*t*_1_ = 280 μs) were collected, with a recovery delay of 1 s. The ^1^H and ^15^N 90° pulse durations were 9.7 and 39.7 µs, respectively. To avoid extended signal averaging which would be required for samples in 96–98% deuterated buffers, the HSQC reference spectra were acquired using fresh samples of the same proteins prepared in a 90% protonated PBS buffer, at pH 7.4 and 37 °C.

### Protein sample preparation

Uniformly ^15^N and ^13^C enriched ubiquitin and OPN samples were prepared as described (Kadeřávek et al. 2018; Platzer et al. 2011). Before dilution by hyperpolarized HDO, protein concentrations were 2 mM in all cases.

## Electronic supplementary material

Below is the link to the electronic supplementary material.

Supplementary file1 (PDF 1103 kb)
