# Supplementary material for: Sensitivity-enhanced three-dimensional and carbon-detected two-dimensional NMR of proteins using hyperpolarized water
Source: J Biomol NMR. 2020 Feb 10;74(2):161–71. doi: 10.1007/s10858-020-00301-5 (PMC7080779; doi:10.1007/s10858-020-00301-5)
Supplement: Supplementary file 1 — Supplementary file1 (PDF 1103 kb) [file 10858_2020_301_MOESM1_ESM.pdf]

Sensitivity-Enhanced Three-Dimensional and Carbon-Detected Two-Dimensional NMR of Proteins using Hyperpolarized Water

Gregory L. Olsen[1,2,], Or Szekely[2], Borja Mateos[3], Pavel Kadeřávek,[4,5] Fabien Ferrage,[5] Robert Konrat[3], Roberta Pierattelli,[6] Isabella C. Felli[6], Geoffrey Bodenhausen[5], Dennis Kurzbach[1,5,*], Lucio Frydman[2]*

[1]*University Vienna, Faculty of Chemistry, Institute for Biological Chemistry, Währinger Straße 38, 1090 Vienna, Austria*
[2]*Department of Chemical and Biological Physics, Weizmann Institute of Science, Rehovot, Israel*
[3] *Department of Structural and Computational Biology, Max Perutz Labs, University of Vienna. ViennaBiocenter Campus 5, 1030, Vienna, Austria.*
[4]*CEITEC–Central European Institute of Technology, Masaryk University, Kamenice 5, CZ-625 00 Brno, Czech Republic*
[5]*Laboratoire des biomolécules, LBM, Département de chimie, École normale supérieure, PSL University, Sorbonne Université, CNRS, 75005 Paris, France*
[6]*Magnetic Resonance Center and Department of Chemistry Ugo Schiff, University of Florence, Via L. Sacconi 6, 50019 Sesto Fiorentino, (FI) – Italy*

* corresponding authors: Gregory.Olsen@univie.ac.at ; Dennis.Kurzbach@univie.ac.at

- Supporting Information -

In most cases, despite extensive signal averaging, the signals in thermal equilibrium spectra were too weak for a reliable determination of the enhancement factor 'ε' (often defined as the ratio between a per-scan signal amplitude in a hyperpolarized spectrum and in a similar spectrum acquired after return of the system to thermal equilibrium.) Therefore, to quantify the signal intensities obtained with hyperpolarized HDO, we report signal-to-noise (SNR) ratios here.

Table 1. SNR values for non-overlapping residues in the hyperpolarization-boosted H^{N}-CON spectrum of ubiquitin (cf. Fig 1 in the main text).

Residue	SNR
5	6
14	9
15	7
17	6
27	6
34	6
73	9

Table 2. SNR values for non-overlapping residues in the hyperpolarization-boosted H^{N}-CON spectrum of OPN (cf. Fig 2a and 2c).

Residue	SNR
60	6
70	7
106	7
125	8
137	7
166	7

212	6
217	6
224	8
225	6
248	6

Table 3. SNR values for non-overlapping residues in hyperpolarization-boosted $^{1}H^{N}$-CON spectrum of OPN in the presence of heparin (cf. Fig 2d).

**Residue**	**SNR**
70	5
87	5
110	7
125	6
154	4
164	5
240	7
246	5
258	7

Table 4. SNR values for residues in the hyperpolarization-boosted HNCO spectrum of ubiquitin (cf. Fig 3).

**Residue**	SNR
3	35
5	35
15	53
17	53
27	52
44	45
57	32
73	22

Table 5. SNR values for non-overlapping residues in the hyperpolarization-boosted HNCO spectrum of OPN (cf. Fig 4).

**Residue**	**SNR**
68	7
72	7
75	8
80	8
107	10
109	27
115	7
134	7
176	6
226	6
234	3
235	8
243	7
245	11
253	1
257	13

260	10
263	6
264	8
266	8

Table 6. SNR values of non-overlapping residues in hyperpolarization-boosted HNCO spectrum of OPN in the presence of heparin (cf. Figs 4b and 4c).

Residue	SNR
70	19
87	4
115	8
141	5
154	8
158	6
164	7
240	5
246	8
258	2
263	4

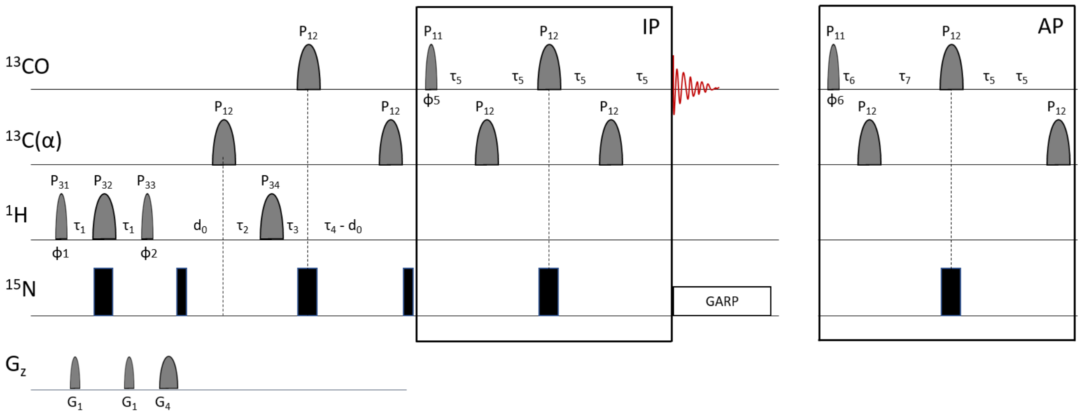

Figure S1. BEST-^{1}H^N-CON pulse sequence [2]. Delays were τ_1 = 137 µs, τ_2 = 1893 µs, τ_3 = 12994 µs, τ_4 = 16501 µs, τ_5 = 8201 µs, τ_6 = 4500 µs, and τ_7 = 11901 µs. Selective ^{1}H 90° and 180° pulses were PC9 (2001.4 µs) and Reburp (1600.2 µs), respectively. Selective ^{13}C 90° and 180° pulses were Q5_sebop (300.2 µs) and Q3_surbop (198.8 µs), respectively. Non-selective ^{15}N 90° pulse durations were 31.5 µs (ubiquitin) or 32.9 µs (OPN). GARP decoupling was applied to ^{15}N during acquisition. The phase cycle was Φ_1 = [x, -x], Φ_2 = [y], Φ_5 = [x], Φ_6 = [-y], and Φ_{rec} = [x, -x]. All other pulses were applied along the x axis (Φ = 0), IPAP was used to suppress CO-Cα splittings during acquisition. Gradients were G_1 (4.6 G/cm, 500 µs) and G_4 (32.85 G/cm, 1 ms), both generated using the Bruker shape library file 'SINE.100'.

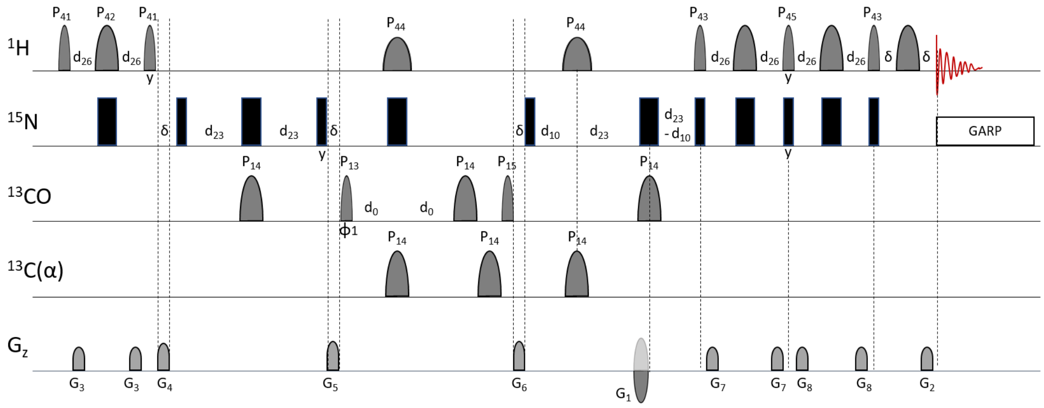

Figure S2. BEST-HNCO pulse sequence[1]. Shaped and hard pulses are shown as oval or square shapes, respectively. The narrow shapes indicate 90° pulses and the broad shapes indicate 180° pulses. Delays were D23 = 14.5 ms, D26 = 2.4 ms, and δ = 1.1 ms. ^{1}H selective pulses were PC9 (P41, 2251 µs) 180° pulses Reburp (P42, 1498 µs), Eburp2 and Eburp2tr (P43 and P45, each 1439 µs), and BIP720,50,20 (P44, 150 µs). ^{13}C selective pulses were G4 and G4tr (P13 and P15, each 308 µs), and Q3 (P14, 210 µs). Non-selective ^{15}N 90° pulse durations were 39.75 µs. GARP decoupling of ^{15}N was applied during acquisition. The phase cycle was Φ_1 = [x, -x], and Φ_{rec} = [x, -x]. All pulses were applied along the x axis (Φ = 0), except when indicated otherwise. Gradients were G_1 (42.8 G/cm, 1 ms), G_2 (4.3 G/cm, 1 ms), G_3 (3.75 G/cm, 300 µs), G_4 (-21.4 G/cm, 1 ms), G_5 (-26.75 G/cm, 1 ms), G_6 (32.1 G/cm, 1 ms), G_7 (-2.68 G/cm, 500 µs), and G_8 (2.68 G/cm, 300 µs), all generated using the Bruker shape library file 'SMSQ10'.

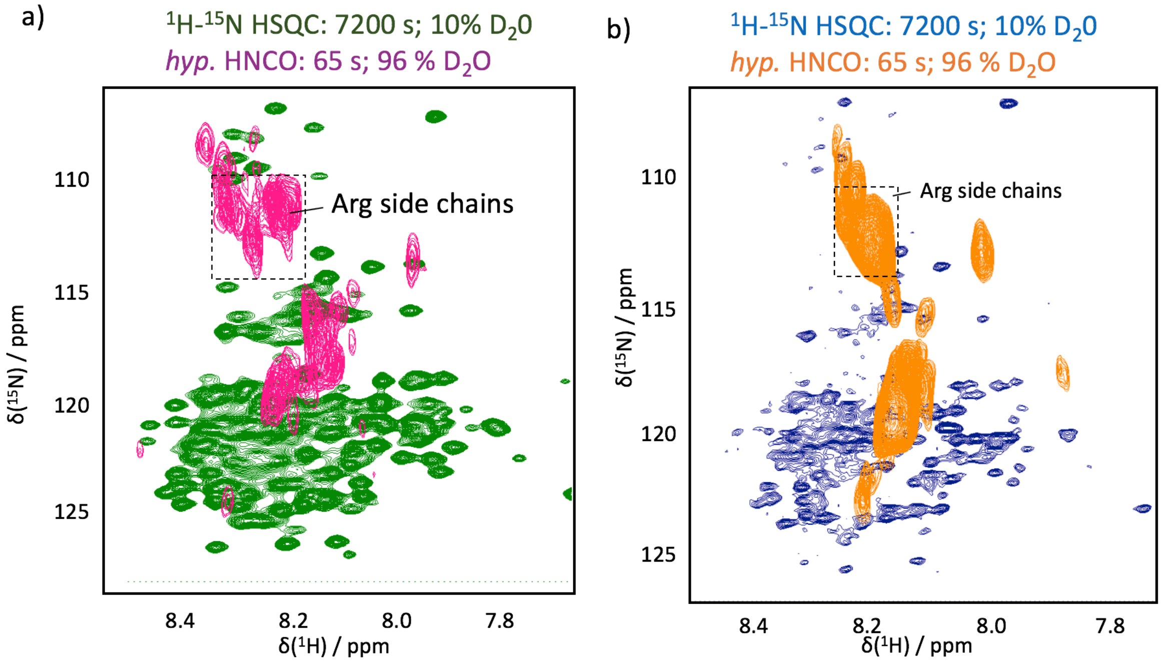

Figure S3. Projections of the hyperpolarized HNCO spectra of OPN in a) the absence and b) the presence of heparin (magenta and orange spectra, respectively). The projections are superimposed on the corresponding thermal equilibrium 2D HSQC NMR spectra.

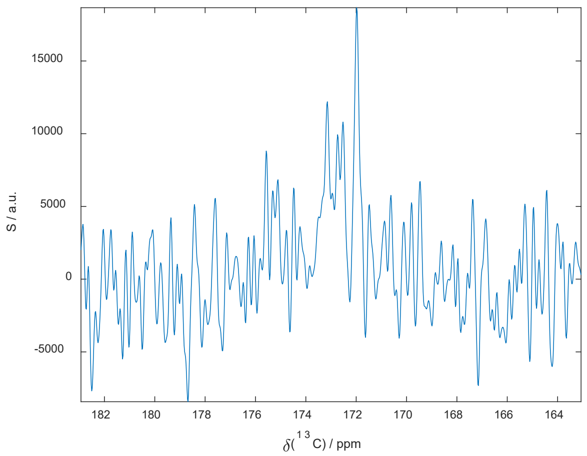

Figure S4. Sum projection of the hyperpolarized $^{1}H^{N}$-CON spectrum of ubiquitin (main text Fig. 1) on the ^{13}C dimension. No peaks could be detected after overnight signal averaging in any of the $^{1}H^{N}$-CON spectra for the same samples in thermal equilibrium.

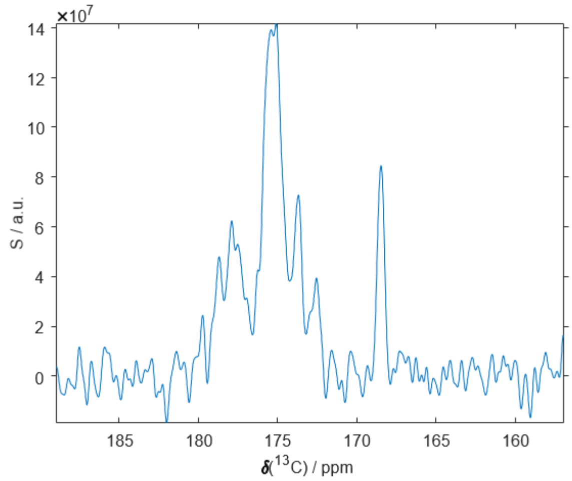

Figure S5. Sum projection of the hyperpolarized $^{1}H^{N}$-CON spectrum of OPN (main text Fig. 2a) onto the ^{13}C dimension.

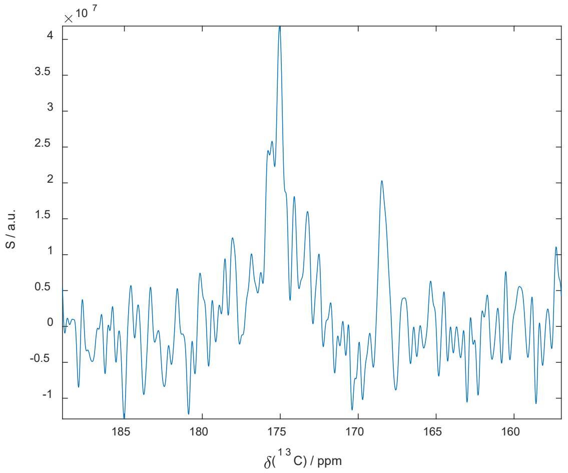

Figure S6. Sum projection of the hyperpolarized $^{1}H^{N}$-CON spectrum of OPN in the presence of 2 eq. heparin (main text Fig. 2d) onto the ^{13}C dimension.

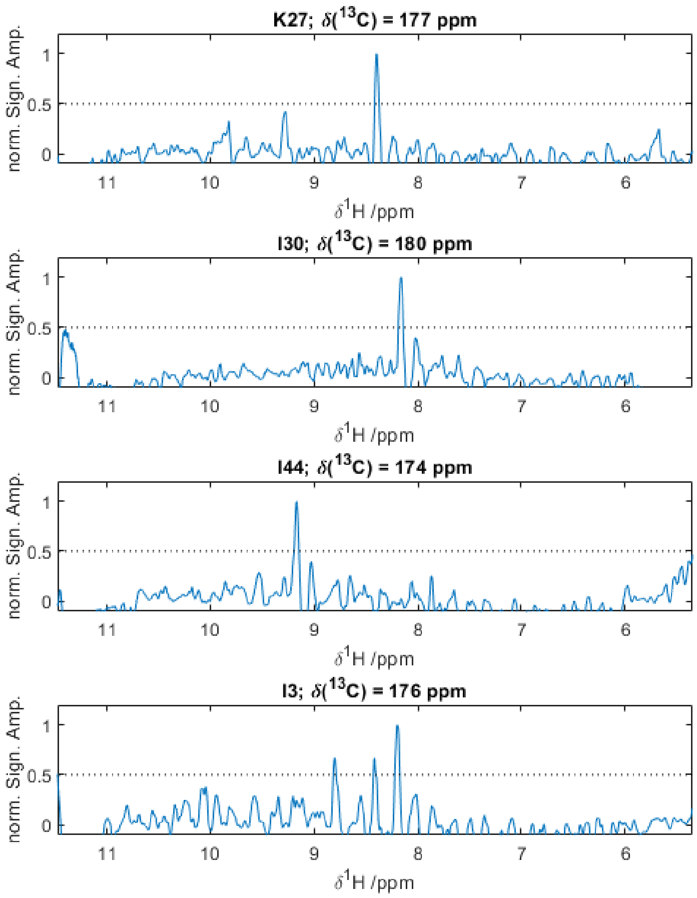

Figure S7. Sum projections of the signals observed in the hyperpolarized HNCO spectrum of ubiquitin (cf. main text, Fig. 3) onto the ^{1}H dimension, for a given ^{13}C resonance frequency. The dashed line indicates the cut-off threshold in the figures of the main text.

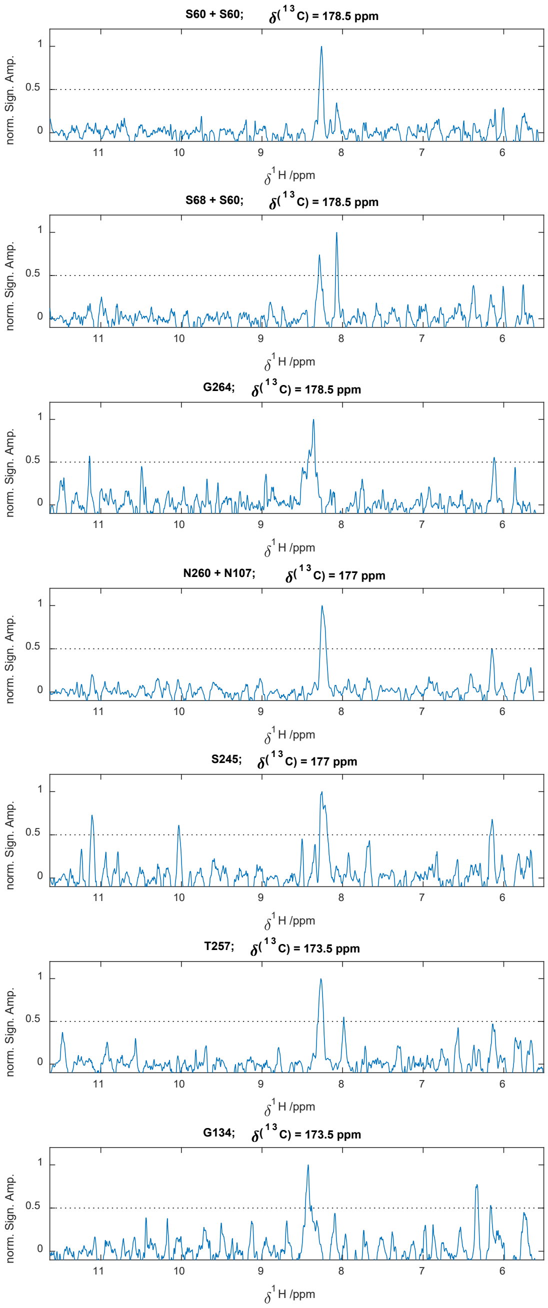

Figure S8. Sum projections of the signals observed in the hyperpolarized HNCO spectrum of OPN (cf. main text, Fig. 4) onto the ^{1}H dimension, for a given ^{13}C resonance frequency. The dashed line indicates the cut-off threshold in the figures of the main text.

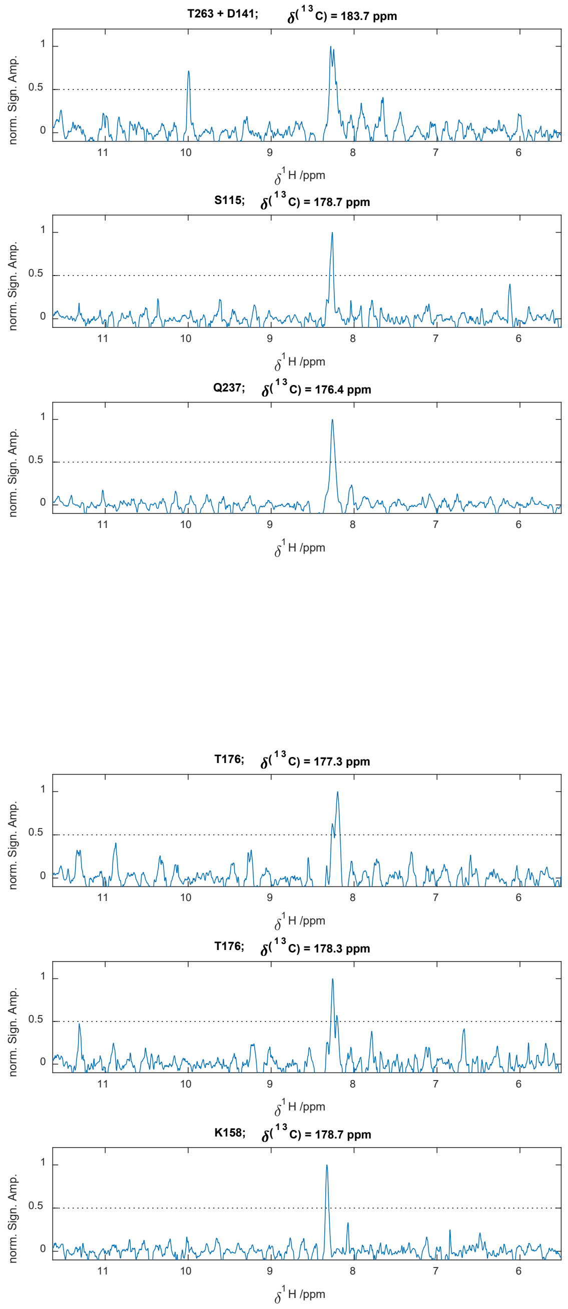

Figure S9. Sum projections of the signals observed in the hyperpolarized HNCO spectrum of OPN in the presence of 2 eq. heparin (cf. main text, Fig. 4) onto the ^{1}H dimension, for a given ^{13}C resonance frequency. The dashed line indicates the cut-off threshold in the figures of the main text.

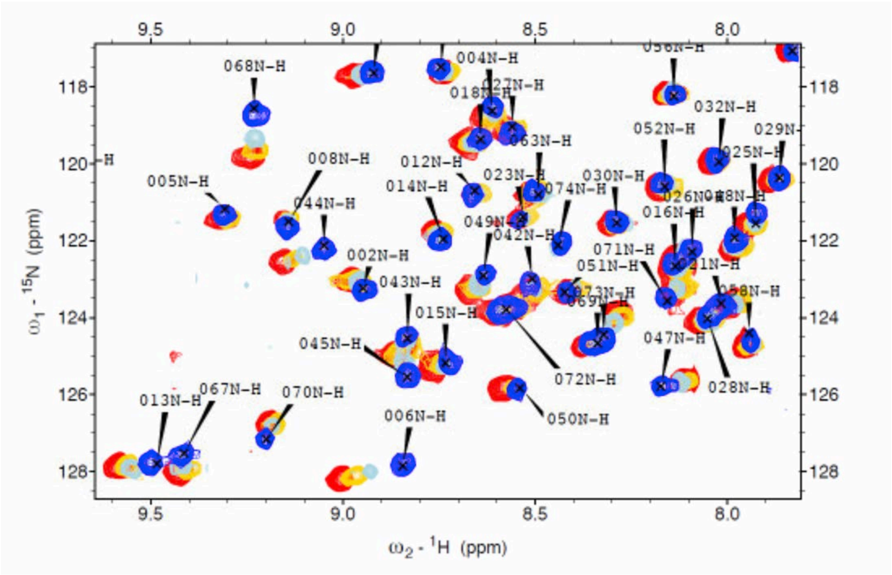

Figure S10. ^1H-^{15}N HSQC pH titration used for assignment of ubiquitin signals at the pH and temperature used in the D-DNP experiments (physiological saline at pH 7.4 and 37˚C). Spectra shown were collected for WT ubiquitin at pH 6, 6.5, 7, 7.4 (blue, light blue, yellow, red, respectively). For many residues significant changes in chemical shifts are observed with increasing pH.

References

1. Lescop, E., Schanda, P. & Brutscher, B. A set of BEST triple-resonance experiments for time-optimized protein resonance assignment. *J Magn Reson* **187**, 163-9 (2007).
2. Gil, S. et al. NMR Spectroscopic Studies of Intrinsically Disordered Proteins at Near-Physiological Conditions. *Angewandte Chemie-International Edition* **52**, 11808-11812 (2013).
